# Semen modulates the differentiation of monocyte-derived dendritic cells towards a tolerogenic profile

**DOI:** 10.1186/1742-4690-9-S2-P199

**Published:** 2012-09-13

**Authors:** F Remes Lenicov, C Rodríguez Rodrigues, C Jancic, J Sabatté, M Cabrini, M Donalson, R Pasqualini, J Geffner, A Ceballos

**Affiliations:** 1 Instituto de Investigaciones Biomedicas en Retrovirus y SIDA, Buenos Aires, Argentina; 2 Instituto Médico Halitus, Buenos Aires, Argentina

## Background

HIV vaccines have not been able to provide immune protection against sexually-transmitted HIV. We hypothesized that semen has an active role in the transmission of HIV by influencing the early events of the immune response. This study analyzed the ability of spermatozoa and seminal plasma (SP) to modulate in vitro the differentiation profile of human monocyte-derived dendritic cells (DC).

## Methods

Spermatozoa were purified by swim-up of semen samples from healthy donors, while SP was obtained by semen centrifugation. DC were obtained by incubating peripheral blood monocytes (>85% purity) with GM-CSF and IL-4 for 5 days, in the absence or presence of spermatozoa (Sp: monocyte ratio 4:1) or SP (dilution 1:5000). DC phenotype was analyzed by flow cytometry while cytokine production was measured by ELISA.

## Results

Differentiation of DC performed in the presence of spermatozoa or SP resulted in a marked reduction of CD1a expreession together with increased expression of CD14. The mean fluorescence intensity for CD1a was: 7464 ± 106, 210 ± 25 and 766 ± 35 (p<0.05 for controls vs spermatozoa- or SP-treated cells, respectively). We also found that spermatozoa and SP significantly (p < 0.05) increased expression of HLA-DR, CD86 and CD80. However, stimulation of treated DC with LPS resulted in reduced production of IL-12p70 or IL-23, but increased secretion of IL-10 (p < 0.01). Moreover, DC differentiated in the presence of SP or spermatozoa induced the expansion of CD25+FOXP3+ T lymphocytes: 5±1% vs 12 ±4% and 9 ±2% CD25+FOXP3+ cells (p<0.05, control vs SP- or spermatozoa-treated cells respectively). Finally, the effect of SP on DC phenotype and IL-12p70 production was partially abrogated by antgonizing prostaglandin-E2 signalling, suggesting a role of prostaglandins as mediators of semen effects.

## Conclusion

Our data support the notion that semen may alter the anti-HIV immune response by inducing a tolerogenic profile in dendritic cells.

